# Effects of Linalyl Acetate on Thymic Stromal Lymphopoietin Production in Mast Cells

**DOI:** 10.3390/molecules23071711

**Published:** 2018-07-13

**Authors:** Phil-Dong Moon, Na-Ra Han, Jin Soo Lee, Hyung-Min Kim, Hyun-Ja Jeong

**Affiliations:** 1 Department of Pharmacology, College of Korean Medicine, Kyung Hee University, Seoul 02447, Korea; pdmoon@khu.ac.kr (P.-D.M.); nrhan@khu.ac.kr (N.-R.H.); mcjin21@naver.com (J.S.L.); 2 Center for Converging Humanities, Kyung Hee University, Seoul 02447, Korea; 3 Department of Food Science & Technology and Research Institute for Basic Science, Hoseo University, Chungnam 31499, Korea

**Keywords:** thymic stromal lymphopoietin, linalyl acetate, nuclear factor-κB, caspase-1, intracellular calcium

## Abstract

Thymic stromal lymphopoietin (TSLP) is an important factor responsible for the pathogenesis of allergic diseases, such as atopic dermatitis and asthma. Because linalyl acetate (LA) possesses a wide range of pharmacological properties, being antispasmodic, anti-inflammatory, and anti-hyperpigmentation, we hypothesized that LA could inhibit TSLP. Therefore, enzyme-linked immunosorbent assay, quantitative polymerase chain reaction, caspase-1 assay, Western blot analysis, fluorescent analyses of the intracellular calcium levels, and the phorbol myristate acetate (PMA)-induced edema model were used to investigate how LA inhibits the production of TSLP in HMC-1 cells. LA reduced the production and mRNA expression of TSLP in HMC-1 cells. LA also inhibited the activation of nuclear factor-κB and degradation of IκBα. PMA plus A23187 stimulation up-regulated caspase-1 activity in HMC-1 cells; however, this up-regulated caspase-1 activity was down-regulated by LA. Finally, LA decreased intracellular calcium levels in HMC-1 cells as well as PMA-induced ear swelling responses in mice. Taken together, these results suggest that LA would be beneficial to treatment of atopic and inflammatory diseases by reducing TSLP.

## 1. Introduction

Atopic dermatitis (AD) is a chronic and relapsing inflammatory skin disease characterized by inflammation, pruritus, and eczematous lesions [1]. Approximately 25% of children and 1–3% of adults in industrialized countries are affected by AD [2]. Moreover, the prevalence of AD has grown greatly in the last ten years, notably in urban areas in developed countries [3]. This increase in prevalence has led to increased healthcare costs as well as decreased quality of life for patients with AD because of its symptoms, which include recurring skin lesions, itchiness, lack of sleep, and dietary limitations [4].

Thymic stromal lymphopoietin (TSLP) is an important factor responsible for the pathogenesis of allergic diseases, such as AD and asthma. Epicutaneous application of house dust mite resulted in the induction of TSLP gene expression in nonlesional skin of patients with AD [5]. Serum levels of TSLP were significantly elevated in AD patients compared with controls [6]. In atopic diseases, such as AD and asthma, mast cells play an important role in addition to keratinocytes and epithelial cells [7]. Indeed, many studies have reported that activation and the number of mast cells are up-regulated in AD models, suggesting the importance of mast cells in AD [8,9,10,11,12].

Caspases are cysteine aspartic proteases with essential roles in programmed cell death; however, caspase-1 is a prototypic inflammatory caspase [13]. Although most caspases generally function in programed cell death, caspase-1 converts interleukin (IL)-1β from an inactive to an active form [14,15,16]. TSLP production was mediated by caspase-1 and nuclear factor (NF)-κB pathway. In addition, NF-κB was down-regulated by pretreatment with caspase-1 inhibitor, indicating that caspase-1 is an upstream factor of NF-κB [17].

**Figure 1:**
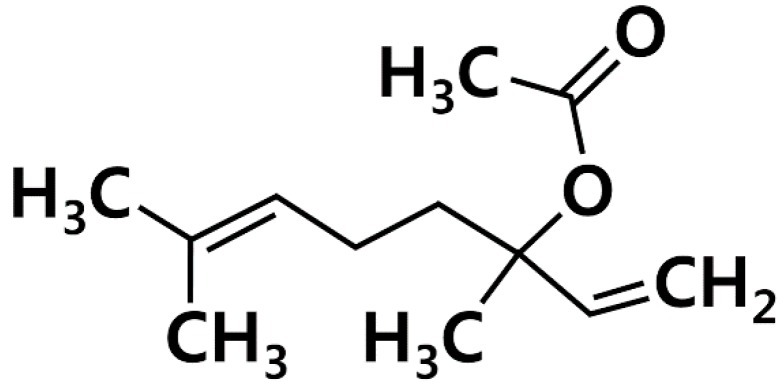
Chemical structure of linalyl acetate (LA).

Linalyl acetate (LA, Figure 1) is one of main constituents of lavender [18]. Because LA possesses a wide range of pharmacological properties, being antispasmodic, anti-inflammatory, and anti-hyperpigmentation [18,19,20], we hypothesized that LA could inhibit TSLP, which is an important factor in allergic diseases. Thus, we investigated how LA affects TSLP production in HMC-1 cells.

## 2. Results

### 2.1. Effect of LA on TSLP Production in HMC-1 Cells

**Figure 2:**
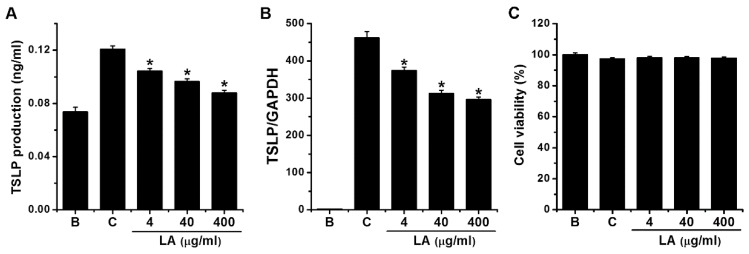
Effects of LA on the production and mRNA expression of TSLP in HMC-1 cells. (**A**) HMC-1 cells (3 × 10^5^) were pretreated with LA at concentrations of 4 to 400 μg/mL for 1 h, and then stimulated by PMA plus A23187 for 7 h. The TSLP levels in the supernatant were assessed using ELISA method. (**B**) HMC-1 cells (1 × 10^6^) were stimulated by PMA plus A23187 for 5 h. The levels of TSLP mRNA expression were measured using qPCR method. (**C**) HMC-1 cells (3 × 10^5^) were pretreated with LA at the doses of 4 to 400 μg/mL for 1 h, and then stimulated with PMA plus A23187 for 7 h. Cell viability was determined with an MTT assay. B, PBS-treated cells; C, PBS-treated, and then PMA plus A23187-stimulated cells. Each datum represents the mean ± S.E.M. of three independent experiments. * *p* < 0.05; significantly different from the control (PBS-treated, and then PMA plus A23187-stimulated cells) value.

To investigate the effects of LA on TSLP production, we stimulated HMC-1 cells with PMA plus A23187 for 7 h, after which ELISA was used to measure the levels of TSLP. Stimulation with PMA plus A23187 stimulated TSLP production in HMC-1 cells; however, this increase was significantly reduced to 0.104 ± 0.002, 0.096 ± 0.002, and 0.088 ± 0.002 by treatment with LA at 4 μg/mL to 400 μg/mL, respectively (Figure 2A, *p* < 0.05). TSLP production levels in the control and blank groups were 0.121 ± 0.003 and 0.074 ± 0.004, respectively. Four μg/mL of LA decreased the TSLP production up to 69.703 ± 4.573%. Treatment with LA alone did not affect the production of TSLP when compared with the blank (PBS-treated cells) group (data not shown). When LA was pretreated at the doses of 4 to 400 μg/mL, the cytotoxicity was not shown by the treatment with LA (Figure 2C).

### 2.2. Effect of LA on TSLP mRNA Expression in HMC-1 Cells

To assess the effects of LA on the TSLP mRNA expression, HMC-1 cells were pretreated with various concentrations (4 to 400 μg/mL) of LA for 1 h before PMA plus A23187 stimulation for 5 h. qPCR analysis revealed that stimulation of HMC-1 cells with PMA plus A23187 up-regulated the TSLP mRNA expression; however, the up-regulated TSLP mRNA expression was reduced by pretreatment with LA (Figure 2B). The relative expression levels of TSLP mRNA at doses of 4 to 400 g/mL were 373.600 ± 9.868, 312.931 ± 9.220, and 296.865 ± 6.640, respectively, while those of the control and blank groups were 461.629 ± 17.307 and 1.933 ± 0.580, respectively. The inhibitory effect of 400 μg/mL of LA was greater than 4 and 40 μg/mL; thus, we assessed the effects of 400 μg/mL of LA in subsequent experiments. 

### 2.3. Effect of LA on NF-κB Activation and IκBα Degradation

**Figure 3:**
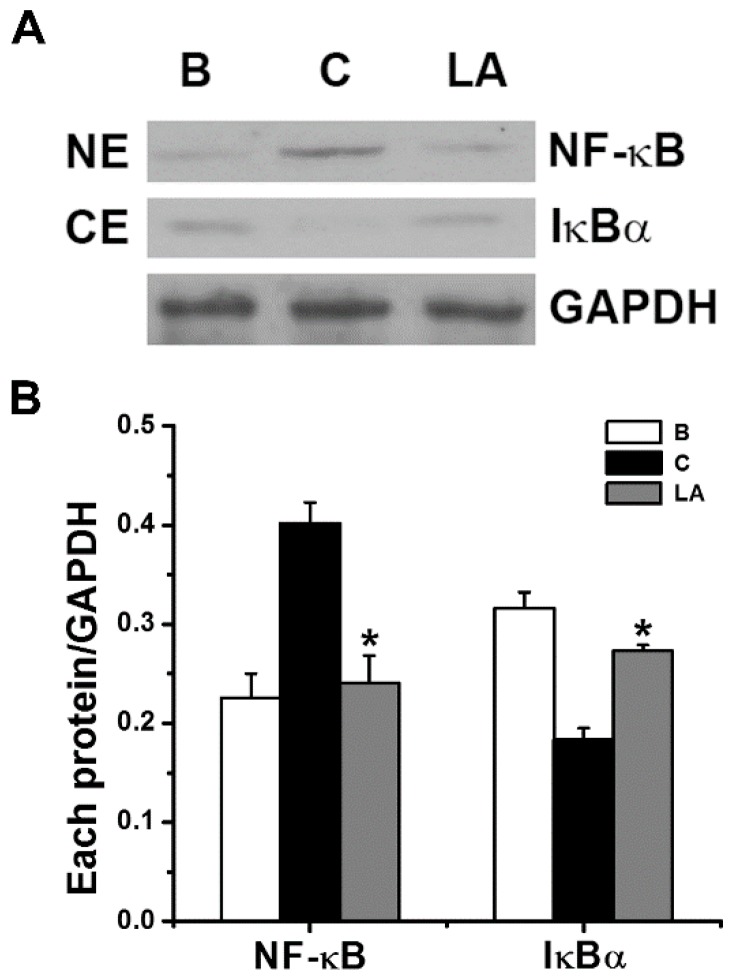
Effects of LA on the activation of NF-κB p65 and degradation of IκBα in HMC-1 cells. (**A**) The HMC-1 cells (5 × 10^6^) were pretreated with LA at the dose of 400 μg/mL for 1 h, and then stimulated by PMA plus A23187 for 2 h. (**B**) Each protein level was quantitated by densitometry. NE, nuclear extract; CE, cytoplasmic extract; B, PBS-treated cells; C, PBS-treated, and then PMA plus A23187-stimulated cells. Each datum represents the mean ± S.E.M. of three independent experiments. * *p* < 0.05; significantly different from the control (PBS-treated, and then PMA plus A23187-stimulated cells) value.

To determine if the inhibitory effects of LA were mediated by NF-κB and IκBα signaling, we analyzed the activation of NF-κB p65 and degradation of IκBα by Western blot analysis. Stimulation of HMC-1 cells with PMA plus A23187 increased the activation of NF-κB in the nuclear extract; however, the increased NF-κB activation decreased in response to pretreatment with LA (Figure 3). The relative intensities of NF-κB in the blank, control, and LA-pretreated groups were 0.226 ± 0.024, 0.401 ± 0.021, and 0.241 ± 0.027, respectively. We examined whether the inhibitory effect of LA was due to its regulation of IκBα degradation because IκBα degradation results in NF-κB activation [21]. Stimulation of HMC-1 cells with PMA plus A23187 increased the degradation of IκBα in the cytoplasmic extract; however, the increased IκBα degradation decreased in response to pretreatment with LA (Figure 3). The relative intensities of IκBα in the blank, control, and LA-pretreated groups were 0.316 ± 0.016, 0.183 ± 0.012, and 0.273 ± 0.006, respectively. 

### 2.4. Effect of LA on Caspase-1 Activation in HMC-1 Cells

**Figure 4:**
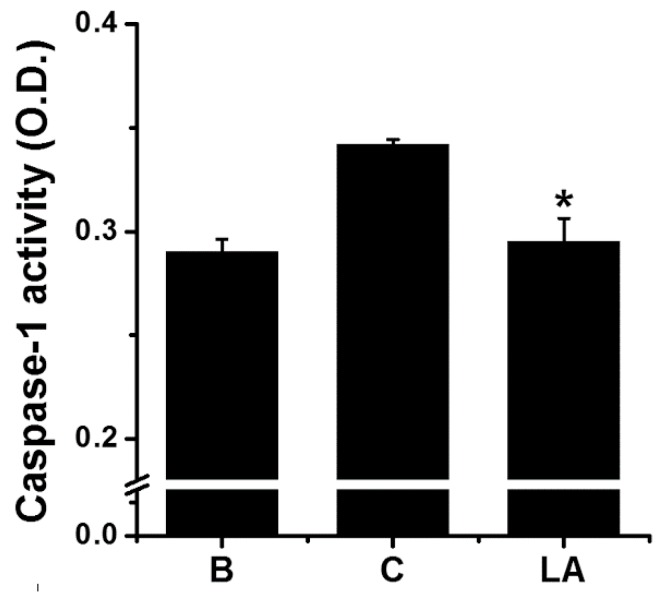
Effect of LA on the caspase-1 activation in HMC-1 cells. HMC-1 cells (5 × 10^6^) were pretreated with LA for 1 h, and then stimulated by PMA plus A23187 for 1 h. Each protein level was quantitated by densitometry. B, PBS-treated cells; C, PBS-treated, and then PMA plus A23187-stimulated cells. Each datum represents the mean ± S.E.M. of three independent experiments. * *p* < 0.05; significantly different from the control (PBS-treated, and then PMA plus A23187-stimulated cells) value.

Caspase-1 activity was measured to investigate whether it mediated the inhibitory effect of LA. Stimulation of HMC-1 cells with PMA plus A23187 increased the activation of caspase-1; however, the increased caspase-1 activation was down-regulated by treatment with LA (Figure 4). The caspase-1 activity values in the blank, control, and LA-pretreated groups were 0.290 ± 0.006, 0.342 ± 0.003, and 0.295 ± 0.011, respectively.

### 2.5. Effect of LA on Intracellular Calcium Level in HMC-1 Cells

**Figure 5:**
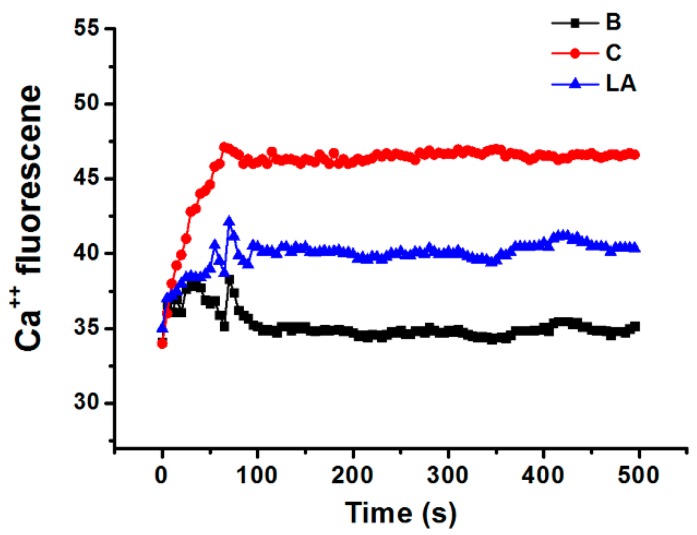
Effect of LA on the level of intracellular calcium in HMC-1 cells. HMC-1 cells were pretreated with LA for 20 min and then stimulated with PMA plus A23187. B, PBS-treated cells; C, PBS-treated, and then PMA plus A23187-stimulated cells.

Calcium is required for caspase-1 activation [22]; thus, we investigated the effects of LA on the level of intracellular calcium in HMC-1 cells. Stimulation of HMC-1 cells with PMA plus A23187 increased the level of intracellular calcium, but these increased intracellular calcium levels were reduced by pretreatment with LA (Figure 5).

### 2.6. Effect of LA on Pro-inflammatory Cytokine Levels in HMC-1 Cells

**Figure 6:**
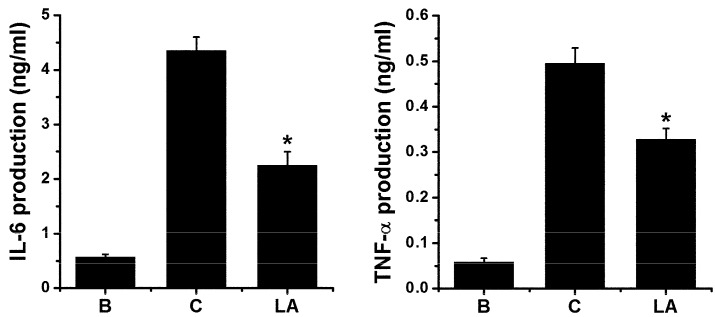
Effect of LA on pro-inflammatory cytokine levels in HMC-1 cells. HMC-1 cells (3 × 10^5^) were pretreated with LA at concentration of 400 μg/mL for 1 h, and then stimulated by PMA plus A23187 for 7 h. The levels of IL-6 and TNF-α in the supernatant were assessed using ELISA method. B, PBS-treated cells; C, PBS-treated, and then PMA plus A23187-stimulated cells. Each datum represents the mean ± S.E.M. of three independent experiments. * *p* < 0.05; significantly different from the control (PBS-treated, and then PMA plus A23187-stimulated cells) value.

To validate the effects of LA, we assessed the levels of pro-inflammatory cytokines, including IL-6 and TNF-α, in HMC-1 cells. Stimulation with PMA plus A23187 increased the production of IL-6 and TNF-α; however, these increased levels were down-regulated by treatment with LA (Figure 6).

### 2.7. Effect of LA in Animal Model

HMC-1 cells do not express FcεRI, are immature, and have low expression of most mast cell markers with the exception of c-Kit and histamine. To overcome these limitations, we tested the effects of LA in an animal model. Topical application of PMA resulted in ear edema in a murine model [23]. In this study, ear thickness increased in response to topical application of PMA, but LA administration ameliorated the ear swelling response (Table 1).

## 3. Discussion

When mast cells are activated by cross-linking of IgE receptors with antigen, protein kinase C (PKC) is activated and intracellular calcium levels are elevated [24,25]. PKC is involved in the activation of NF-κB and production of cytokines [24]. To reproduce this process, we used PMA to activate PKC and A23187 to elevate intracellular calcium levels in this study. Our previous study revealed that PMA plus A23187 stimulation increases cytokine TSLP production in HMC-1 cells [17], and skin lesions of patients with AD have been shown to express high levels of TSLP [26]. Moreover, Oyoshi et al. [27] reported that TSLP promotes skin inflammatory reactions in a mouse model of AD, while the well-known anti-inflammatory drug, dexamethasone, suppressed TSLP expression in an AD mouse model [28]. The results of the present study revealed that LA decreased the production and mRNA expression of TSLP in HMC-1 cells (Figure 2). Thus, we assume that LA might be beneficial in atopic and inflammatory diseases. As shown in Figure 2, the levels of mRNA and protein were quite different. Okayama et al. [29] reported that secreted TSLP was rapidly degraded by proteases secreted by mast cells. Similarly, the results of the present study also showed that the elevation in the amount of secreted TSLP protein was much lower than the increase in mRNA. Thus, we presuppose that a considerable amount of the secreted TSLP would be degraded by proteases that were secreted by mast cells.

Dexamethasone is a well-known anti-inflammatory drug that has shown inhibitory effects against various inflammatory diseases, including pancreatitis and asthma, that occur through the down-regulation of NF-κB activation [30,31]. Lee and Ziegler [32] reported that TSLP gene regulation was controlled by NF-κB. Moreover, the production and mRNA expression of TSLP were found to be mediated by NF-κB in mast cells [17]. In the present study, LA reduced NF-κB activation and IκBα degradation (Figure 3). Furthermore, Shen et al. [33] reported that NF-κB is a key transcription factor in TSLP production. Thus, we assumed that LA could inhibit TSLP through down-regulation of NF-κB activation in HMC-1 cells and would be beneficial to treatment of various inflammatory diseases like dexamethasone. 

Typically, pro-inflammatory stimulation results in the activation of caspase-1 [34]. Several studies have shown that the activation of caspase-1 is induced by pro-inflammatory stimulation, such as PMA plus A23187 [35,36,37]. The results of the present study revealed that LA inhibited the activation of caspase-1 (Figure 4); therefore, it can be assumed that LA inhibits TSLP production and mRNA expression through blocking of caspase-1 activation in mast cells. 

Calcium is required for caspase-1 activation [22], and 1,2-bis(2-aminophenoxy)ethane-N,N,N0,N0-tetraacetic acid acetoxymethyl ester (BAPTA-AM) is an intracellular calcium chelator. Pretreatment with BAPTA-AM was shown to decrease the activation of caspase-1 in PMA plus A23187-stimulated cells [38], while the results of the present study showed that LA inhibits the level of intracellular calcium in HMC-1 cells (Figure 5). Taken together, these results provide evidence that LA inhibits TSLP through blockage of intracellular calcium/caspase-1/NF-κB signaling in HMC-1 cells (Figure 6).

**Figure 7:**
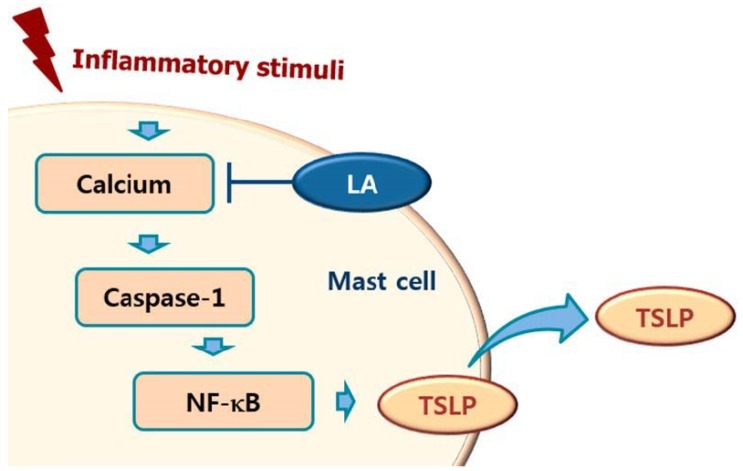
Schematic diagram of proposed regulation of TSLP by LA. Upon receipt of inflammatory stimuli, the level of intracellular calcium increased, and increased intracellular calcium resulted in the activation of caspase-1, and then the activated caspase-1 induced NF-κB activation in mast cells. Finally, the activated NF-κB induced the production of TSLP from mast cells. In this study, LA inhibited the production of TSLP by down-regulating the intracellular calcium/caspase-1/NF-κB signal cascade in mast cells.

LA treatment inhibited the production of TSLP through down-regulation of the NF-κB/IκBα pathway in HMC-1 cells. Well-known cytokines including IL-6 and TNF-α have also been shown to be regulated by the NF-κB/IκBα pathway [39,40]. As expected, LA treatment inhibited the production of IL-6 and TNF-α in HMC-1 cells (Figure 7). Thus, we validated our results through the inhibition of IL-6 and TNF-α. 

In the present study, we used HMC-1 cells to test the effects of LA on the production of TSLP. However, HMC-1 cells do not express FcεRI. Furthermore, HMC-1 cells are immature and have low expression of most mast cell markers, with the exception of c-Kit and histamine. The results of the present study demonstrated that LA administration decreased the ear swelling induced by PMA in mice (Table 1). From the results of in vivo study, we presuppose that the anti-inflammatory effects of LA could be easily translated to the human disease.

In conclusion, we showed that LA inhibited TSLP production and mRNA expression through blockade of the caspase-1/NF-κB pathway. In addition, LA decreased the intracellular calcium levels. Taken together, the results of this study suggest that LA would be helpful to cure atopic and inflammatory diseases through inhibition of TSLP.

## 4. Materials and Methods

### 4.1. Materials

We purchased Power SYBR^®^ Green PCR master mix from Applied Biosystems (Warrington, UK). Recombinant human standard (TSLP), TSLP antibody, and caspase-1 assay kits were obtained from R&D Systems (Minneapolis, MN, USA). Linalyl acetate, A23187, dimethyl sulfoxide, phorbol myristate acetate (PMA), and avidin-peroxidase were acquired from Sigma-Aldrich Corp. (St. Louis, MO, USA). NF-κB p65, IκBα, and GAPDH antibodies were purchased from Santa Cruz Biotechnology (Santa Cruz, CA, USA). Recombinant human standards (IL-6 and TNF-α), IL-6 and TNF-α antibodies, and TMB substrate were obtained from Pharmingen (San Diego, CA, USA).

### 4.2. Cells

Since TSLP was produced in similar levels by HMC-1 cells and bone marrow-derived mast cells [41], we investigated the effects of LA in the human mast cell line, HMC-1. The HMC-1 cells were cultured in IMDM containing 100 μg/mL streptomycin, 100 U/mL penicillin, and 10% heat-inactivated fetal bovine serum at 37 °C in the presence of 5% CO_2_.

### 4.3. MTT Assay

To assess the cell viability, MTT assay was conducted as previously described [42]. First, we seeded HMC-1 cells (3 × 10^5^) in 24-well plates, which were subsequently incubated with MTT (5 mg/mL) for 4 h at 37 °C under 5% CO_2_ and 95% air. Next, 1 mL of dimethyl sulfoxide was added to dissolve the MTT formazan, after which 200 μL of supernatant was removed into a new 96-well microplate. Finally, we read the plate at 540 nm.

### 4.4. Cytokines Assay

Enzyme-linked immunosorbent assay (ELISA) was used to assess the levels of TSLP, IL-6, and TNF-α from the culture supernatant, as previously described [43,44]. Sandwich ELISA for TSLP, IL-6, and TNF-α was performed in 96-well microplates. Capture antibodies for TSLP (0.8 μg/mL), IL-6 (4 μg/mL), and TNF-α (6 μg/mL) were incubated in the plates overnight at 4 °C. The plates were then washed with PBS containing 0.05% Tween-20 (PBST) and blocked with PBS containing 1% BSA, 5% sucrose and 0.05% NaN_3_ for 1 h. After washing, samples and each standard were incubated in the plates at room temperature (RT) for 2 h. Detection antibodies for TSLP (0.4 μg/mL), IL-6 (2 μg/mL), and TNF-α (2 μg/mL) were incubated at RT for 2 h. After washing, streptavidin-peroxidase was added and the plates were incubated at 37 °C for 30 min. After additional washes, TMB substrate (Pharmingen) was added. Finally, the absorbance of the plates at 405 nm was measured using a colorimetric microplate reader.

### 4.5. Quantitative Polymerase Chain Reaction (qPCR)

qPCR was performed using Power SYBR Green PCR master mix, and the detection of mRNA was conducted with an ABI StepOne real-time PCR system (Applied Biosystems, Foster City, CA, USA), as previously described [45,46]. Briefly, total RNA (1 μg) was heated at 75 °C for 5 min, then chilled on ice. cDNA was subsequently prepared from the total RNA with a cDNA synthesis kit at 42 °C for 1 h. Next, PCR was conducted using the following primers: TSLP (5′ CCC AGG CTA TTC GGA AAC TCA G3′; 5′ CGC CAC AAT CCT TGT AAT TGT G3′); GAPDH (5′ TCG ACA GTC AGC CGC ATC TTC TTT3′; 5′ ACC AAA TCC GTT GAC TCC GAC CTT3′). The PCR conditions were as follows: initial denaturation at 95 °C for 10 min, followed by 40 cycles of denaturation at 95 °C for 15 s and annealing at 60 °C for 30 s with melting curve analysis. The level of the target mRNA was normalized to the level of the GAPDH and compared with that of the control. All data were analyzed using the ΔΔCT method.

### 4.6. Caspase-1 Assay

The activation level of caspase-1 was assessed using a caspase-1 assay kit according to the manufacturer’s instructions. Briefly, HMC-1 cells were lysed with lysis buffer for 1 h, then centrifuged at 15,000× *g* for 20 min. Next, 50 μL of supernatant was incubated with 50 μL of 2× reaction buffer (containing 10 mM DTT and 200 μM YVAD-pNA substrate) in 96-well microplates for 2 h. Finally, the plates were read at 450 nm.

### 4.7. Preparation of Nuclear and Cytoplasmic Extracts

Nuclear and cytoplasmic extracts were prepared as described previously [40,47]. Briefly, HMC-1 cells were lysed with 50 μL of cold hypotonic buffer (10 mM Hepes/KOH, 2 mM MgCl_2_, 0.1 mM EDTA, 10 mM KCl, 1 mM DTT, and 0.5 mM PMSF, pH 7.9). After 20 min, 2.5 μL of 10% Nonidet P (NP)-40 was added and the cells were centrifuged at 15,000× *g* for 5 min at 4 °C. The supernatant was then used as the cytoplasmic extract. The pellets of nuclei were gently resuspended in 40 μL of cold saline buffer (50 mM HEPES/KOH, 50 mM KCl, 300 mM NaCl, 0.1 mM EDTA, 10% glycerol, 1 mM DTT, and 0.5 mM PMSF, pH 7.9) and left on ice for 20 min. Next, samples were centrifuged at 15,000× *g* for 20 min at 4 °C, after which the supernatant was used as the nuclear extract.

### 4.8. Western Blot Analysis

The cell lysates were heated in sample buffer (62.5 mM Tris-HCl, pH 6.8, 2% sodium dodecyl sulphate (SDS), 20% glycerol, and 10% 2-mercaptoethanol) at 95 °C for 5 min, then briefly cooled on ice, as previously described [40,48,49]. Next, samples were centrifuged at 15,000× *g* for 5 min, after which proteins were separated by 10% SDS-polyacrylamide gel electrophoresis and transferred to nitrocellulose paper. The membranes were blocked with 5% skim milk in PBS for 2 h, then incubated with primary (NF-κB, IκBα, and GAPDH; 1:500 dilution) and secondary (mouse anti-rabbit IgG-HRP and bovine anti-mouse IgG-HRP; 1:5000 dilution) antibodies. Finally, protein bands were visualized using an enhanced chemiluminescence solution purchased from Amersham Co. (Newark, NJ, USA).

### 4.9. Fluorescent Measurements of the Intracellular Calcium Level

To assess the changes in the level of intracellular calcium with time, HMC-1 cells (1 × 10^5^) were pretreated with Fura-2/AM in IMDM containing 10% FBS for 30 min, and then harvested as previously described [50,51]. After washing twice with medium containing extracellular calcium chelator EGTA (0.5 mM), the cell suspension (1 × 10^5^ cells) was seeded into a 96-well plate and pretreated with LA for 20 min. Next, HMC-1 cells were stimulated with PMA plus A23187 for 5 min. The plate was then analyzed at 440 nm (excitation 360 nm) in a spectrofluorometer. Because the intracellular calcium levels increased steeply within 100 s and then remained at similar levels from 100 s to 1000 s, we measured intracellular calcium levels until 500 s.

### 4.10. PMA-Induced Ear Edema in Mice

We tested the effects of LA in an animal model as described by Fujie et al. [23]. Brielfy, 20 μL of PMA (0.1 mg/mL in acetone) was applied to both the inner and outer surfaces of the ears of mice (total 40 μL/ear) 1 h after LA administration. The thickness of the ear was then measured using a micrometer before PMA application, then measured again 6 h later.

### 4.11. Statistical Analysis

To analyze the results, IBM SPSS Statistics 23 was used. All results were expressed as the means ± standard errors of the mean (S.E.M.). Differences between groups were identified by independent *t*-tests and ANOVA with Tukey’s post hoc test. *p* < 0.05 was considered to indicate statistical significance.

## Figures and Tables

**Table 1:** Effect of LA on PMA-induced ear edema in mice.

Treatment	Dose (mg/kg)	Pre-Thickness (mm)	Post-Thickness (mm)	Increase (mm)
B	-	0.3358 ± 0.0041	0.3390 ± 0.0035	0.0032 ± 0.0016
C	-	0.3335 ± 0.0062	0.4375 ± 0.0102	0.1040 ± 0.0072
LA	400	0.3342 ± 0.0066	0.3905 ± 0.0038	0.0563 ± 0.0049 *

LA was administered orally with sonde 1 h before the application of PMA. After 1 h, edema was induced by topical application of PMA (0.1 mg/mL) dissolved in acetone. PMA was applied in a volume of 20 μL to both the inner and outer surfaces of the ears of mice (total 40 μL/ear). B, vehicle (acetone)-applied mice; C, PMA-applied mice. Each datum represents the mean ± S.E.M. with n = 3 mice per group. * *p* < 0.05; significantly different from the control (PMA-applied mice) value.
